# Developmental variation in DNA methylation in poplar (*Populus trichocarpa*)

**DOI:** 10.1186/1753-6561-5-S7-P177

**Published:** 2011-09-13

**Authors:** Kelly Vining, Kyle R Pomraning, Larry Wilhelm, Henry D Priest, Cathleen Ma, Ruoqing Zhu, Elizabeth Etherington, Matteo Pellegrini, Todd Mockler, Michael Freitag, Steven H Strauss

**Affiliations:** 1 Department of Forest Ecosystems and Society, Oregon State University, Corvallis, OR 97331, USA; 2 Molecular and Cellular Biology Program, Dept. of Biochemistry and Biophysics, Center for Genome Research and Biocomputing, Oregon State University, Corvallis, OR 97331, USA; 3 Department of Botany and Plant Pathology, Center for Genome Research and Biocomputing, Oregon State University, Corvallis, OR 97331, USA; 4 Deptartment of Molecular, Cell and Developmental Biology, University of California, Los Angeles, Los Angeles, CA, 90024, USA; 5 Dept. of Biochemistry and Biophysics, Center for Genome Research and Biocomputing, Oregon State University, Corvallis, OR 97331, USA; 6 Department of Forest Ecosystems and Society, Center for Genome Research and Biocomputing, Oregon State University, Corvallis, OR 97331, USA

## 

Using methylated DNA immunoprecipitation followed by high-throughput sequencing (MeDIP-seq), we analyzed DNA methylation patterns in the *P. trichocarpa* genome in relation to four biological processes: bud dormancy and release, mature organ maintenance, in vitro organogenesis, and methylation suppression. Here, we report results from Illumina sequencing of nine sampled tissues, each representing 1 to 2 biological replicates. We sequenced 26M – 97M reads per tissue type, and validated our MeDIP-seq results using bisulfite sequencing of selected targets.

Unique MeDIP-seq reads covered ~30-60% of genome space at an average depth of 4 to 12 reads/nucleotide. Transposons and other repeat elements were enriched within the methylated fraction of the genome. The pattern of gene model methylation showed higher methylation at promoters, in the middle of coding regions, and 3’ to ends of genes, similar to that observed in other plant and animal species. Numbers of methylated genes identified varied widely by tissue type.

**Figure 1:**
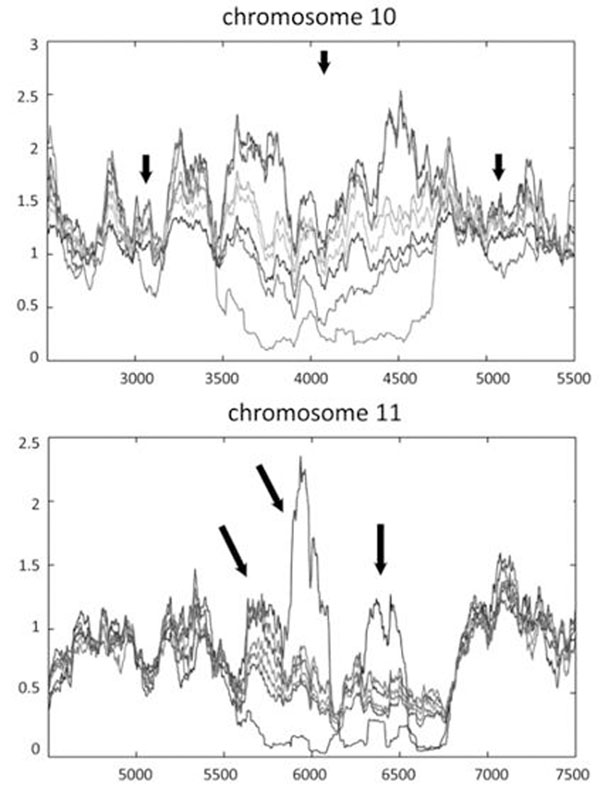
**Two examples of section of poplar chromosomes showing tissue-specific methylation.** Each line represents one tissue type, including roots, buds, xylem, phloem, leaves, male catkins, and female catkins. Arrows point to regions showing strong tissue-specific differentiation. X-asix is 1 Kb tiled windows. Y-axis is RPKM.

We produced summary data for genome methylation in *P. trichocarpa*, including the distribution of methylation across chromosomes (Fig. 1) and in and around genes. The intensity of methylation was highly heterogeneous within and between chromosomes. One-third of the genome, analyzed as 1 kb tiled windows, was differentially methylated among tissues. An example of a chromosomal region with differential methylation is shown in Fig. 2. Promoter methylation was more frequent than gene body methylation. Male catkins differed from the other tissues in that gene body methylation was more prevalent than promoter methylation, and two transposable element categories that were methylated in all other tissues were unmethylated in male catkins. We also analyzed the association of methylation intensity to gene expression data from an existing microarray study of the same tissues. At a whole genome scale, both promoter-methylated and body-methylated genes had lower expression than unmethylated genes. We will report on our continued studies of tissue methylation/expression relationships.

We have developed a customized genome browser (Gbrowse version 1.69), compatible with the most recent (v2.2) *P. trichocarpa* genome assembly, at which our data can be explored: http://poplar.cgrb.oregonstate.edu.

